# Clinicopathological Significance of FOXO4 Expression and Correlation with Prx1 in Head and Neck Squamous Cell Carcinoma

**DOI:** 10.1155/2021/5510753

**Published:** 2021-05-17

**Authors:** Yunping Lu, Yajun Shen, Lingyu Li, Min Zhang, Min Wang, Lihua Ge, Jing Yang, Xiaofei Tang

**Affiliations:** Beijing Institute of Dental Research, Beijing Key Laboratory, Beijing Stomatological Hospital & School of Stomatology, Capital Medical University, No. 4 Tiantanxili, Dongcheng District, Beijing 100050, China

## Abstract

**Objective:**

Forkhead box O 4 (FOXO4), a key albumen in the forkhead box O (FOXOs) family, plays crucial roles as a tumor suppressor in the cancer development. In our previous study, Peroxiredoxin1 (Prx1) promoted the development of oral cancer and was predicted to bind to FOXO4. The aim of this study was to investigate the clinicopathological significance of FOXO4 expression and its potential mechanism in head and neck squamous cell carcinomas (HNSCC).

**Methods:**

The function of FOXO4 correlation with HNSCC prognosis was analyzed via ONCOMINE, UALCAN, Human Protein Atlas, and cBioPortal. The expression of FOXO4 was detected in Prx1 silenced CaL27 and SCC9 cell lines by Western blot. FOXO4 protein expression was observed via immunohistochemistry (IHC) and the binding of Prx1 to FOXO4 measured by Duolink analysis in a 4-nitro-quinoline-1-oxide- (4NQO-) induced tongue carcinogenesis model in Prx1^+/+^ and Prx1^+/−^ mice.

**Results:**

By the analysis of Bioinformation Databases, there was a significant interaction of FOXO4 down expression to clinical tumor stages and pathological grades in the patients with HNSCC. Reduced mRNA and protein expression of FOXO4 were found to be significantly correlated with the poor overall survival (OS) of HNSCC patients. FOXO4 expression is negatively related to Prx1 significantly in HNSCC tissues. By employing a 4NQO-induced oral carcinogenesis mouse model, we confirmed that FOXO4 expression was reduced in 4NQO-induced squamous cell carcinoma (SCC) tongue tissues compared with those in normal tissues. Prx1 knockdown resulted in the upregulation of FOXO4 expression in the SCC tissues and CaL27 and SCC9 cell lines. Furthermore, the interaction of Prx1 with FOXO4 was observed in mouse tongue tissues by Duolink analysis.

**Conclusion:**

FOXO4 plays an important role in the development of HNSCC. The lower expression of FOXO4 is significantly correlated with the shorter OS in patients with HNSCC. FOXO4 is negatively regulated via interaction with Prx1. FOXO4 could be a potential molecular target for the treatment and prognosis of HNSCC.

## 1. Introduction

Head and neck squamous cell carcinoma (HNSCC) is the sixth malignancy with 700,000 new cases and 380,000 deaths rising annually globally. The 5-year overall survival (OS) rate of patients with oral cancer was less than 50% [1, 2]. The development of HNSCC is a complex and multistep process, which is affected by tumor biology and external stimulating factors. Studies have shown that the risk factors of HNSCC include tobacco, alcohol, betel nut, and human papillomavirus (HPV) [3, 4]. In addition, dietary factors and lifestyle have increasingly been shown to be risk factors [5]. Several genes and signal pathways underlying the development of HNSCC have been explored. The ubiquitin conjugating enzyme E2 (UBE2C) plays an oncogenic role in HNSCC, and UBE2C overexpression predicts a shorter survival [6]. Patients with cyclooxygenase-2 (COX-2) overexpression suffered a worse overall survival rate [7]. It reported that the PI3K/AKT/PTEN pathway and COX-2 signaling pathway may contribute to early tumorigenesis and angiogenesis in HNSCC [7, 8]. The unclear mechanism of pathogenesis seriously affects clinical treatment and survival rate of HNSCC. It is urgent to find typical molecular markers for improving HNSCC prognosis.

FOXOs, a fork head transcription factor family with four members (FOXO1, 3, 4, and 6), play key roles as tumor suppressors providing resistance to oxidative stress and inducing cell cycle G1/S arrest and apoptosis during tumorigenesis [9, 10]. Increasing evidence has shown that dysregulation of FOXO4 expression could accelerate tumor progression including cervical, colorectal, pancreatic, and lung cancers [11]. Until now, the underlying functions and mechanisms of FOXO4 in HNSCC remain elusive.

Peroxiredoxin1 (Prx1), an important antioxidant enzyme, is overexpressed in various malignant tumors. Reportedly, Prx1 is strongly linked to the development of malignant tumors through regulating oxidative stress, cell proliferation, differentiation, invasion, and migration [12]. Our previous studies have showed that Prx1 can promote oral carcinogenesis and metastasis [13, 14]. Prx1 is predicted to bind to FOXO4 in dysplastic oral keratinocyte (DOK) cells according to proteomic analysis [15]. In this study, we analyzed the clinicopathological significance of FOXO4 expression based on the public databases. The potential mechanism of FOXO4 was investigated in a 4-nitro-quinoline-1-oxide- (4NQO-) induced tongue carcinogenesis model in Prx1^+/+^ and Prx1^+/−^ mice and OSCC cell lines.

## 2. Methods

### 2.1. ONCOMINE Database

ONCOMINE (http://www.oncomine.org) is a cancer microarray database used for the genome-wide expression analyses [16]. The differential expression of FOXO4 in HNSCC analysis was directly adopted using ONCOMINE online analysis tools. The data type is mRNA and cut-off of *p* value and fold change as follows: *p* = 0.01, fold change = 1.5, and gene rank = 10%.

### 2.2. UALCAN

UALCAN (http://ualcan.path.uab.edu), which is explored based on the Cancer Genome Atlas (TCGA) level RNA-seq and clinical data from 31 cancer types, was used for in-depth analyses of gene expression. The FOXO4 mRNA expression in normal and HNSCC tissues was carried out from the database. Furthermore, we analyzed the expression in different groups of HNSCC patients, including a patient's age, individual cancer stages, and histological subtypes. The prognosis of HNSCC was analyzed followed with UALCAN [17].

### 2.3. Human Protein Atlas (HPA)

The Human Protein Atlas (http://www.proteinatlas.org) collected human protein-coding gene information including their RNA and protein expression and localization [18]. Immunohistochemistry analysis (IHC) images of FOXO4 protein expression in clinical specimens of patients with HNSCC and normal tissues were obtained from the HPA database, and the expression of FOXO4 protein was investigated to determine the prognosis of HNSCC.

### 2.4. cBioPortal Analysis

cBioPortal (http://www.cbioportal.org) is an open website resource for exploring, visualizing, and analyzing multidimensional cancer genomics data [19]. Alteration and the associated overall survival rate in HNSCC were obtained via cBioPortal. The track displays an overview of genetic alterations of FOXO4 per sample.

### 2.5. LinkedOmics Database Analysis

Identification of FOXO4-associated genes and gene expression correlation analysis were performed via the LinkCompare module of LinkedOmics (http://www.linkedomics.org/login.php) [20]. The Pearson correlation coefficient was used to statistically analyze. We screened out the differentially expressed genes that correlated with FOXO4 in the HNSCC patients. The results were shown in the form of volcano.

### 2.6. Cell Culture

The cell lines Cal27 (CRL-2095) and SCC9 (CRL-1629) were purchased from ATCC and cultured in high-glucose Dulbecco's modified Eagle's medium (DMEM High Glucose, Gibco, USA) and Ham's F12, containing 10% fetal bovine serum (FBS, Gibco, USA), 1% penicillin/streptomycin (Beyotime, China), and 400 ng/mL hydrocortisone (Beyotime, China). Cells were transfected with negative control (NC) and shPrx1 (5′-CTCTTGACTTCACCTTTGTGT-3′, GeneChem, China) which were selected using 1 *μ*g/mL puromycin (Sigma, USA).

### 2.7. Western Blot

Whole cell lysates were denatured in a 5 × SDS loading buffer at 100°C for 10 min. Proteins were separated by SDS-PAGE (Bio-Rad, USA), semidry electrophoretic transferred to PVDF membrane (Bio-Rad, USA), and analyzed using the specified antibodies and an ECL detection system (Bio-Rad, USA) with VCL used as a loading control. The primary antibodies were incubated as follows: anti-Prx1 (1 : 8000, Abcam), anti-FOXO4 (1 : 500, CST), and anti-VCL (1 : 1000, CST).

### 2.8. Animal Studies

All animal experiments were approved by the Animal Ethical and Welfare Committee of Beijing Stomatological Hospital (Approval No. KQYY-201809-001) and were carried out according to the Beijing Municipality on the Review of Welfare and Ethics of Laboratory Animals guidelines. All methods were carried out in accordance with relevant guidelines and regulations.

Wild type (Prx1^+/+^, C57BL/6) mice and Prx1 knockdown (Prx1^+/−^, C57BL/6) mice, 6-8 weeks old and weighing 25-30 g, were established in our laboratory as described previously [21]. This study uses the method of our previous work, and the method description partly reproduces the wording [14, 22]. Prx1^+/+^ (*n* = 15,8 males and 7 females) and Prx1^+/−^ (*n* = 15,8 males and 7 females) mice were divided randomly into control groups (*n* = 5) and 4NQO-treated groups (*n* = 10), respectively. In this study, we employed the tongue carcinogenesis model induced by 4NQO under the guidance of our previous study [14]. All the mice were euthanized, and tongues were removed; then FOXO4 expression was analyzed via IHC based on histopathological changes. Histological changes in the tongue mucosa were validated by two pathologists including normal mucosa, epithelial hyperplasia, epithelial dysplasia, and SCC according to the World Health Organization classification of head and neck tumors (4th Edition, 2017).

### 2.9. Immunohistochemistry Analysis (IHC)

After antigen repair and blockage of endogenous peroxidases, all sections were incubated with anti-FOXO4 (1 : 50, Abcam) overnight at 4°C and secondary antibody (Maixin, China) was applied at 37°C for 30 min. Finally, slides were evaluated using the brown DAB precipitate (Maixin, China) and stained with hematoxylin. Under the microscope (Olympus BX61, Japan), three regions were randomly selected from all sections for MOD (MOD = integral optical density/measurement area) analysis.

### 2.10. Protein-Protein Docking

Docking of FOXO4 (PDB ID:3L2C) with Prx1 (PDB ID:4xcs) was performed using ZDOCK from Discovery Studio (DS) 2016 [23]. The best docked pose was selected based on the evaluation of the binding interface residue by the ZRANK. The docking structure was further visualized by DS.

### 2.11. Duo Link Analysis

Duolink proximity ligation assay (PLA) was used to detect the interaction between Prx1 and FOXO4 in mouse slides. The PLA allows for protein detection under endogenous protein expression levels with high sensitivity and specificity in situ. The experimental steps were performed as described previously [15].

### 2.12. Statistical Analysis

Statistical analysis was performed using the SPSS 20.0 (IBM Corp., Armonk, NY, USA). Differences between variables were analyzed via Student's *t*-test or a one-way analysis of variance (ANOVA). The continuous variables were analyzed using the Mann–Whitney *U* test or Kruskal-Wallis test with Dunn's posttest. The rank correlation among the different variables was assessed with the Pearson correlation coefficient test. *p* value < 0.05 was considered statistically significant.

## 3. Results

### 3.1. Transcriptional Level of FOXO4 in Patients with HNSCC

**Figure 1:**
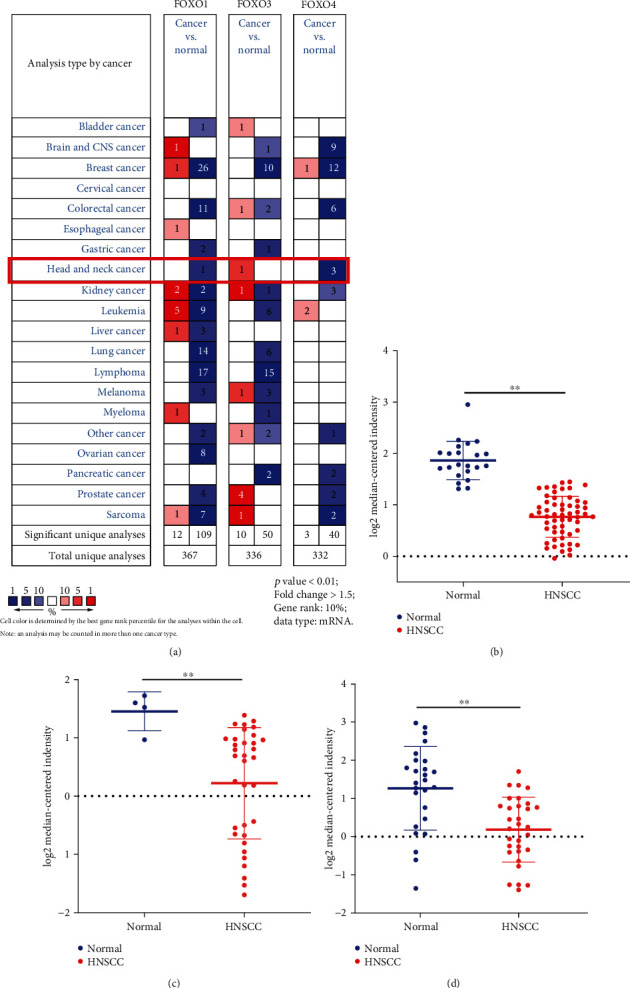
mRNA expression of FOXO4 from ONCOMINE data. (a) mRNA expression of FOXO4 in HNSCC samples. (b–d) Validation of mRNA expression level of FOXO4 in three datasets. Cut-off *p* = 0.01, fold change > 1.5, Gene Rank = 10%; ^∗∗^*p* < 0.01, data type: mRNA.

The transcriptional levels of FOXO4 in HNSCC and normal samples were compared via ONCOMINE databases. The mRNA expression of FOXO4 was downregulated significantly in patients with HNSCC in three different datasets (Figure 1(a)). In the Peng Head-Neck dataset (oral cavity squamous cell carcinoma), FOXO4 was downregulated in tumor tissues compared with normal tissues and the fold change was -2.049 (*p* = 6.56*e* − 19 < 0.01, Figure 1(b)), while it was found to have a 2.351-fold mRNA decrease in FOXO4 in the Cromer Head-Neck dataset (*p* = 1.48*e* − 4 < 0.01, Figure 1(c)) and 2.114-fold downregulation in FOXO4 mRNA expression in the Estilo Head-Neck dataset from tongue SCC (*p* = 8.29*e* − 5 < 0.01, Figure 1(d)).

### 3.2. Correlation between FOXO4 Expression and the Clinicopathological Characteristics of Patients with HNSCC

**Figure 2:**
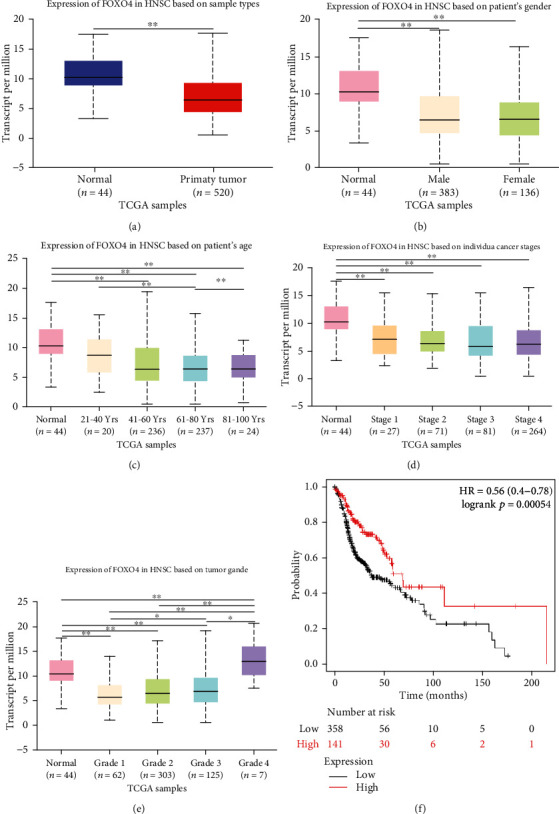
Relationship between mRNA expression of FOXO4 and cancer stages of patients with HNSCC (UALCAN). (a) Expression of FOXO4 in normal and HNSCC samples. (b) Expression of FOXO4 in individuals of gender. (c) Expression of FOXO4 in normal individuals or any age in HNSCC patients aged 21-40, 41-60, 61-80, or 81-100 yr. (d) Expression of FOXO4 in normal individuals or in HNSCC patients in stage 1, 2, 3, or 4. (e) Expression of FOXO4 in normal individuals or HNSCC patients with grade 1, 2, 3, or 4 tumors. Grade 1: well differentiated (low grade); grade 2: moderately differentiated (intermediate grade); grade 3: poorly differentiated (high grade); grade 4: undifferentiated (high grade). (f) Prognostic value of mRNA expression of FOXO4 in HNSCC patients (Kaplan-Meier Plotter). ^∗^*p* < 0.05; ^∗∗^*p* < 0.01.

Next, the relationship between FOXO4 mRNA expression and clinicopathological parameters of patients with HNSCC were analyzed via UALCAN (data based on TCGA). As shown in Figure 2(a), the mRNA expression of FOXO4 was significantly downregulated in HNSCC patients. There was no significant difference of FOXO4 expression between male and female HNSCC patients, although there was a significant decrease, respectively, compared to normal patients (Figure 2(b)). FOXO4 expression was significantly lower in HNSCC patients than normal controls in the individual analysis based on age (Figure 2(c)). The FOXO4 mRNA expression was remarkably correlated with the patients' HNSCC clinical stage (Figure 2(d)). The mRNA level of FOXO4 was significantly related to tumor pathological grade. The lower expression of FOXO4 tended to correlate with a more undifferentiated pathological grade (Figure 2(e)). Additionally, the lower mRNA expression of FOXO4 significantly contributed to the shorter OS of HNSCC patients via Kaplan-Meier Plotter (Figure 2(f), *p* < 0.01).

**Figure 3:**
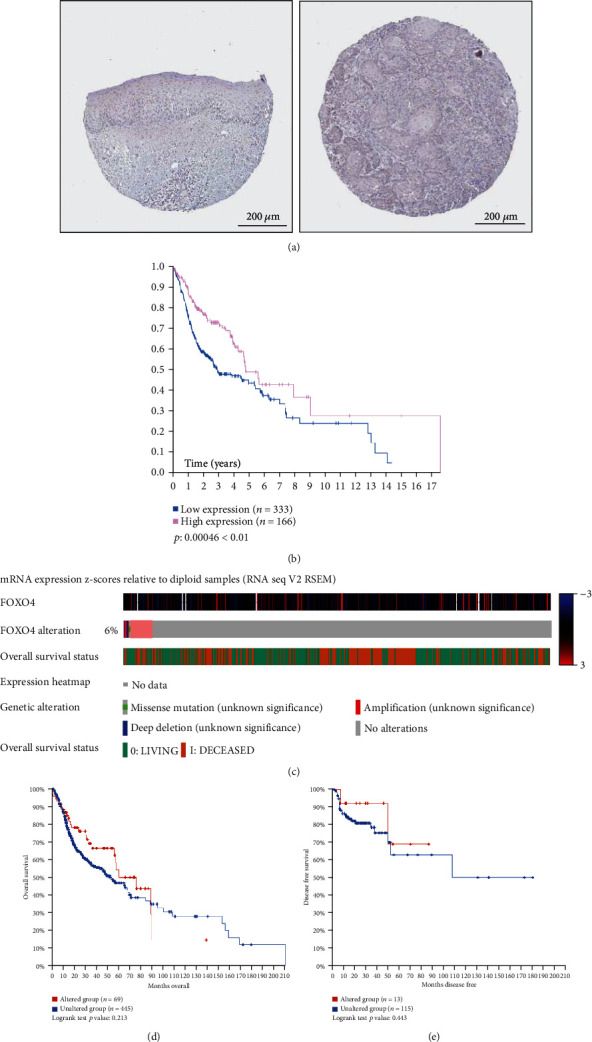
Immunohistochemistry images of FOXO4 in normal tissues or HNSCC tissues (Human Protein Atlas) and genetic alteration of FOXO4 in HNSCC patients (cBioPortal). (a) Low protein expressions of FOXO4 in normal tissues and FOXO4 proteins were not detected in HNSCC tissues. (b) Survival analysis of protein expression of FOXO4 in HNSCC patients (Kaplan-Meier Plotter, ^∗∗^*p* < 0.01). (d) Low FOXO4 mutation rate (6%) in HNSCC patients. (e) Associated OS and (f) DFS were no significantly difference between the altered group and unaltered group of HNSCC patients (*p* > 0.05).

Representative immunohistochemistry images of FOXO4 in normal and HNSCC tissues were obtained from the HPA. Lower protein expressions of FOXO4 were found in normal tissues, whereas no expression was detected in HNSCC tissues (Figure 3(a)). Moreover, as shown in Figure 3(b), a superior long-term survival rate was observed in the HNSCC patients presenting a higher FOXO4 protein expression level (*p* < 0.01).

Furthermore, we analyzed the genetic alteration of FOXO4 in HNSCC patients and their associations with OS and DFS. Genetic alteration was found in 33 of the 515 patients with HNSCC. As shown in Figure 3(c), the FOXO4 alteration rate was 6% in HNSCC patients. There was no correlation between genetic mutation of FOXO4 with shorter OS (Figure 3(d), *p* > 0.05) or shorter DFS (Figure 3(e), *p* > 0.05) of HNSCC patients based on the Kaplan-Meier plot and log-rank test.

### 3.3. FOXO4 Expression Is Negatively Associated with Prx1 in Patients with HNSCC

**Figure 4:**
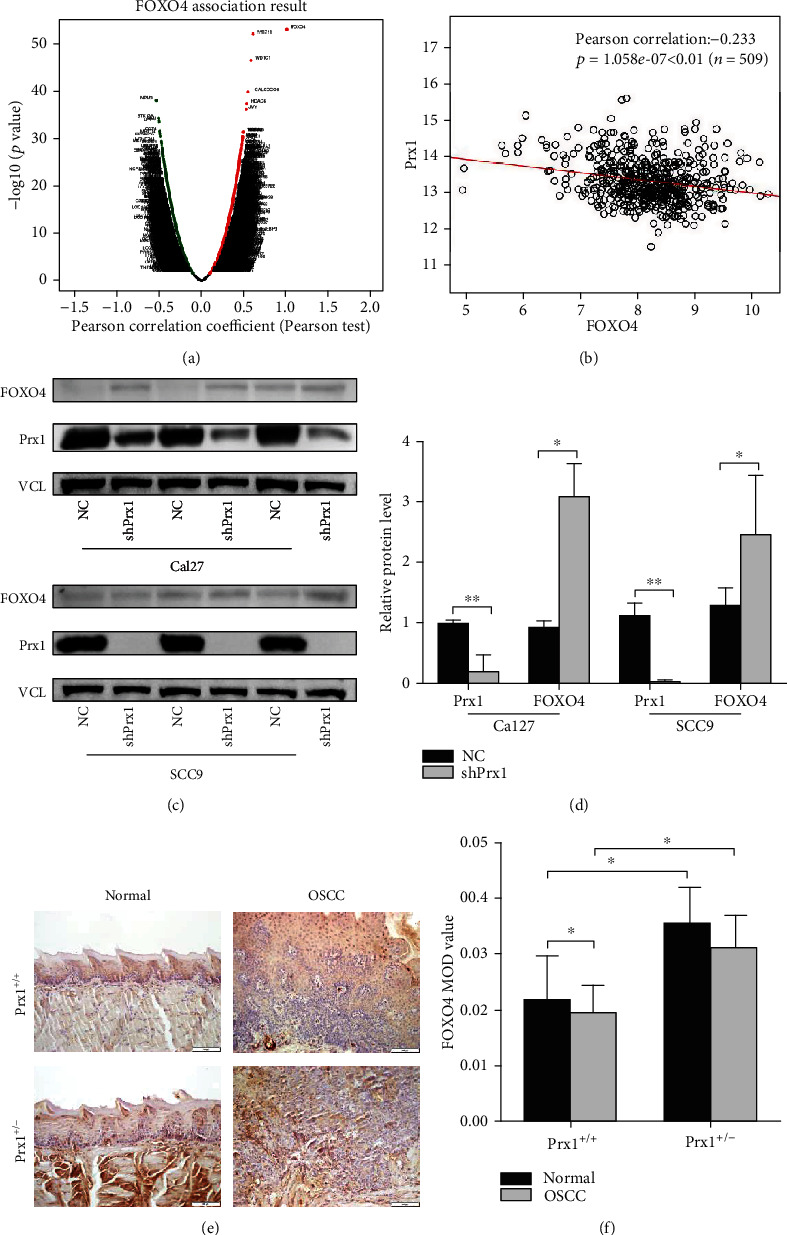
FOXO4 coexpression genes in HNSCC (LinkedOmics). (a) Genes positively and negatively correlated with FOXO4 in HNSCC were shown as volcano plot. Red indicates positively correlated genes, and green indicates negatively correlated genes. (b) The Pearson test was used to analyze correlation between FOXO4 and Prx1 in HNSCC. (c, d) The expression of FOXO4 in shPrx1 Cal27 and shPrx1 SCC9 cells. The relative levels of protein were normalized to VCL used as an internal control. Values presented as the means ± SD (^∗^*p* < 0.05; ^∗∗^*p* < 0.01). (e) FOXO4 protein levels measured by IHC in tongue tissues from Prx1^+/+^ and Prx1^+/−^ mice. (f) FOXO4 MOD value in mouse tongue tissues. (magnification: 200x, the value represents the mean ± SD, and ^∗^*p* < 0.05).

As shown in the volcano plot (Figure 4(a)), there were 2,596 genes (red dots) and 3,050 genes (green dots) showing significant positive and negative correlations with FOXO4 (FDR < 0.01). The results indicated the broad effect of FOXO4. In Figure 4(b), FOXO4 expression showed a negative association with Prx1 which plays an active role in the progression of HNSCC (Pearson correlation: -0.223, *p* < 0.01).

In the present study, Western blotting was used to confirm the expression of FOXO4 in oral cancer cell lines CaL27 and SCC9. The results showed that the level of FOXO4 expression was low, and Prx1 knockdown resulted in a significant increase in FOXO4 expression in Cal27 and SCC9 cells (*p* < 0.05, Figures 4(c) and 4(d)).

### 3.4. Prx1 Knockdown Upregulates FOXO4 in Mouse Tongue Tissues

To further assess the role and relationship between FOXO4 and Prx1 in oral cancer, we detected FOXO4 protein expression in 4NQO induced tongue carcinogenesis model in Prx1^+/+^and Prx1^+/−^ mice. FOXO4 expression in the SCC tissues was significantly decreased when compared with those in normal tissues of Prx1^+/+^ mice (Figures 4(e) and 4(f), *p* < 0.05). However, in Prx1^+/−^ mice, compared with the normal tissues, the decrease of FOXO4 expression in SCC tissues had no significant difference (Figures 4(e) and 4(f), *p* > 0.05). In Prx1^+/−^ mice, FOXO4 expression levels were increased in normal and SCC tissues compared with those in Prx1^+/+^ mice (Figures 4(e) and 4(f), *p* < 0.05). This further confirmed that FOXO4 was reduced in SCC tissues, and Prx1 knockdown upregulated FOXO4 expression.

### 3.5. FOXO4 Interacts with Prx1 in Mouse Tongue Tissues

**Figure 5:**
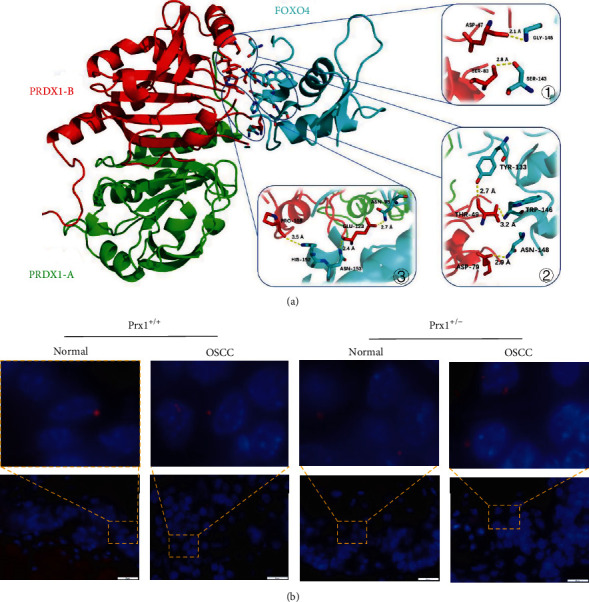
Interaction between Prx1 and FOXO4 (ZDOCK and PLA of Duolink analysis). (a) Structure of Prx1 bound to FOXO4 and FOXO4-Prx1 interaction plot. Prx1(A) is shown in green, Prx1(B) is shown in red, and FOXO4 is shown in cyan. Hydrogen bonds are shown as yellow dashed lines. The images were constructed using Discovery Studio (DS) 2016. (b) Interactions of Prx1 with FOXO4 were detected in tongue tissues from Prx1^+/+^ and Prx1^+/−^ mice. PLA signal is red, and the nuclei is blue (magnification: 1000x).

The docking score of FOXO4 and Prx1 was 84.82, and every possible FOXO4-Prx1 binding pose was scored using an energy-based scoring function. The ZDOCK docking results indicated that FOXO4-Prx1(B) exhibited surface complementarity in the interface area while no force existed between FOXO4 and the A chain of Prx1 (Figure 5(a)). Then, the H-bond interactions of FOXO4-Prx1(B) were analyzed by DS. Prx1(B) Asp47 and Ser83 formed H-bonds with FOXO4 Gly145 and Ser143 (Figure 5(a) ①) and Prx1(B) Thr-49 and Ser83 formed H-bonds with FOXO4 Gly145 and Ser143 (Figure 5(a) ②). Prx1(B) Pro108 and Glu123 formed H-bonds with FOXO4 His152 and Asn153 (Figure 5(a) ③).

To investigate the mechanism of FOXO4 in HNSCC, the Duolink PLA was used to detect protein interactions *in situ*. Accordingly, FOXO4 and Prx1 heterodimers were visualized as red punctate dots with microscopy in the 4NQO-induced tongue tissues (Figure 5(b)), suggesting that FOXO4 may interact directly with Prx1 to affect the progression of OSCC.

## 4. Discussion

HNSCC is one of the most common malignant tumors that threatens human life and health, with OSCC considered the most frequent malignant tumors in head and neck with a higher risk of metastasis and recurrence [24, 25]. Although the efficacy of surgical resection combined with chemoradiotherapy in improving outcomes among patients with HNSCC has been well described, the survival rate for patients with advanced stages remains poor. What is more, there are no effective markers for early diagnosis which limits the prevention and treatment of oral cancer. Identification of accuracy, reproducibility, and reliability biomarkers can lead to early detection of HNSCC and guide management. Some studies showed p16 is a surrogate marker of HPV infection in HNSCC and p16 expression predicts better clinical outcomes [26, 27]. In addition, PET imaging and PDL1 are established diagnostic biomarkers that are clinically used in HNSCC [27]. As a tumor suppressor gene, p53 mutation and overexpression in OSCC resulted in the poor prognosis [28]. HNSCC is a multistep process involving multiple oncogenes and tumor suppressor genes including p16, cyclin D1, and p53 [29].

FOXOs, belonging to the fork head transcription factor family, are involved in cell fate including cell cycle arrested, cell proliferation, and apoptosis [10, 25]. Some studies have shown that FOXOs is negatively regulated by the phosphoinositide PI3k/AKT signaling pathway, which promotes cytoplasmic sequestration of FOXO and results in FOXO inactivation in several cancers. Recent experimental and clinical studies have investigated that FOXO3 and FOXO4 play an important role in the progression of breast cancer, pancreatic cancer, and other cancers [30, 31]. FOXO4 expression has been reported to be decreased in renal cancer and bladder cancer [32]. During tumorigenesis, the ability of FOXO4 to eliminate reactive oxygen species (ROS) was found to be downregulated by the PI3K/Akt pathway, which leads to an increase of intracellular ROS level. However, increased ROS production can produce stress-activated kinases and c-Jun N-terminal kinases (JNK) to enhance the FOXO4 activity and capabilities [10].

In this study, the mRNA level of FOXO4 expression was obtained and analyzed using the Ualcan (based on TCGA) and ONCOMINE databases to explore the relationship between FOXO4 expression and the clinical significance in HNSCC patients. The results showed that FOXO4 expression was significantly lower in HNSCC tissues than in normal tissues, and the lower FOXO4 expression was associated with lower OS rate among these patients. The loss of FOXO4 expression was associated with the degree of tumor differentiation and clinical stage based on the data from TCGA. FOXO4 mRNA expression was remarkably correlated with the patients' individual HNSCC clinical stage and tumor pathological grade. The lower expression of FOXO4 tended to correlate with a more undifferentiated pathological grade. The lowest mRNA expression of FOXO4 was observed at grade 3 instead of grade 4 (Figure 2(e)), possibly due to the small number of patients at grade 4 (7 patients). In summary, the data revealed that the transcriptional levels and protein expressions of FOXO4 are significantly correlated with clinicopathological parameters in HNSCC patients. It suggested that FOXO4 can be prognostic biomarkers for survivals of HNSCC patients.

In our previous studies, we found that Prx1 plays vital roles via possibly binding to FOXO4 in the human dysplastic oral keratinocyte (DOK) cells based on mass spectrometry and bioinformatics analyses [15]. Reportedly, Prx1 is mainly located in the cytoplasm and nucleus with the main antioxidative function [33]. Prx1 prevents cells from oxidative stress with clearing ROS [12]. Our previous study revealed that the expression of Prx1 was increased in human oral leukoplakia (OLK) and OSCC tissues. In the 4NQO-induced tongue OLK animal model, Prx1 knockdown increased cell apoptosis and inhibited the oral carcinogenesis. Furthermore, with the OSCC xenograft model, we found Prx1 knockdown significantly inhibited tongue cancer metastasis rates and EMT progression [13, 14]. Some studies showed that FOXO4 is also an antioxidant mainly located in the nucleus [34]. FOXO4 can cease oxidative stress, induce apoptosis, and influence senescence by PI3K/AKT, Ras-MEK-ERK, IKK, and AMPK pathways [10]. Prx1 can regulate FOXO3 directly as a binding partner [35].

In the present study, Prx1 negatively regulated FOXO4 expression in HNSCC patients based on the cBioPortal analysis. We confirmed that Prx1 knockdown increased the expression of FOXO4 in the Cal27 and SCC9 cells. Similarly, Prx1 silencing induced upregulation of FOXO4 expression in 4NQO-induced SCC tongue tissues. Duolink analysis showed that Prx1 interacted with FOXO4 in mouse tongue tissues. Based on these data, we concluded that under the oxidative stress conditions, Prx1 interacts with FOXO4 and negatively regulates its expression. Subsequently, Prx1 promotes cytoplasmic sequestration of FOXO4 and results in some FOXO4 inactivation. Their coordination action promotes the development of oral cancer. The potential mechanisms of FOXO4 in the progression of HNSCC need to be further studied.

## 5. Conclusions

FOXO4 plays an important role in the development of HNSCC. The lower expression of FOXO4 is correlated with the shorter OS in the patients with HNSCC. Prx1 interacts with FOXO4 and negatively regulated its expression. The findings indicated that FOXO4 may be a potential molecular target for the treatment and prognosis in HNSCC.

## Data Availability

All data generated or analyzed during this study are included in this paper. Some of the data supporting this study are from previously reported studies and datasets from Bioinformation Databases, which have been cited.
